# Stakeholder Perceptions of a Web-Based Physical Activity Intervention for COPD: A Mixed-Methods Study

**DOI:** 10.3390/jcm12196296

**Published:** 2023-09-29

**Authors:** Stephanie A. Robinson, Stephanie L. Shimada, Samantha K. Sliwinski, Renda S. Wiener, Marilyn L. Moy

**Affiliations:** 1 Center for Healthcare Organization and Implementation Research (CHOIR), VA Bedford Healthcare System, Bedford, MA 01730, USA; stephanie.shimada@va.gov; 2 The Pulmonary Center, Boston University School of Medicine, Boston, MA 02118, USA; renda.wiener@va.gov; 3 Department of Health Law, Policy and Management, Boston University School of Public Health, Boston, MA 02118, USA; 4 Division of Health Informatics and Implementation Science, Department of Population and Quantitative Health Sciences, University of Massachusetts Medical School, Worcester, MA 01655, USA; 5 Center for Healthcare Organization and Implementation Research (CHOIR), VA Boston Healthcare System, Boston, MA 02130, USA; samantha.sliwinski@va.gov; 6 Pulmonary, Critical Care, and Sleep Medicine Section, VA Boston Healthcare System, Boston, MA 02130, USA; marilyn.moy@va.gov; 7 Department of Medicine, Harvard Medical School, Boston, MA 02115, USA

**Keywords:** COPD, technology, implementation, mixed methods, physical activity, pulmonary rehabilitation

## Abstract

Technology-based physical activity interventions have been shown to be efficacious in chronic obstructive pulmonary disease (COPD), though their potential impact has not been fully realized due to ineffective implementation. We used a convergent, parallel mixed-methods design to identify patient- and provider-facing barriers and facilitators to implementing a rigorously studied web-based physical activity intervention for COPD. Quantitative surveys (based on the unified theory of acceptance and use of technology; range 1 (poor usability)—5 (high usability)) and semi-structured interviews (guided by the practical robust implementation and sustainability model) assessed the perspectives of 15 patients and 15 health care providers. The patients and providers rated the usability of the intervention as high (median = 5.0, IQR = 1.0). For both patients and providers, the main facilitators included: the potential high impact of the intervention on patient health, the usefulness of the intervention for unmet clinical needs, and the perceived ease of use of the intervention. The main barriers identified were digital literacy and its fit with current clinical workflows. Implementation efforts may benefit from supporting patients’ use of the website and developing strategies to integrate referrals to the intervention and the monitoring of patients into current clinical infrastructures.

## 1. Introduction

Chronic obstructive pulmonary disease (COPD) is a leading cause of death worldwide [1,2,3]. COPD is characterized by breathlessness and benefits from active disease management, including pharmacological and nonpharmacological treatments or interventions [3]. As part of the nonpharmacological treatments, exercise and physical activity is significantly associated with a reduced risk of acute disease exacerbations, hospital admissions, and all-cause mortality [4,5,6,7,8,9].

In-person exercise programs, such as pulmonary rehabilitation (PR), are the standard of care and significantly improve outcomes in COPD patients [10]. However, a multitude of barriers prevent those who would benefit from these in-person programs from attending, including external barriers (e.g., time required to travel to the site and lack of transportation) and internal barriers (e.g., lack of perceived benefit, safety concerns, and motivation) [11,12]. As such, advances in remote, technology-delivered interventions have the potential to overcome a number of these barriers and deliver disease self-management support directly to the patient [13,14].

**Figure 1:**
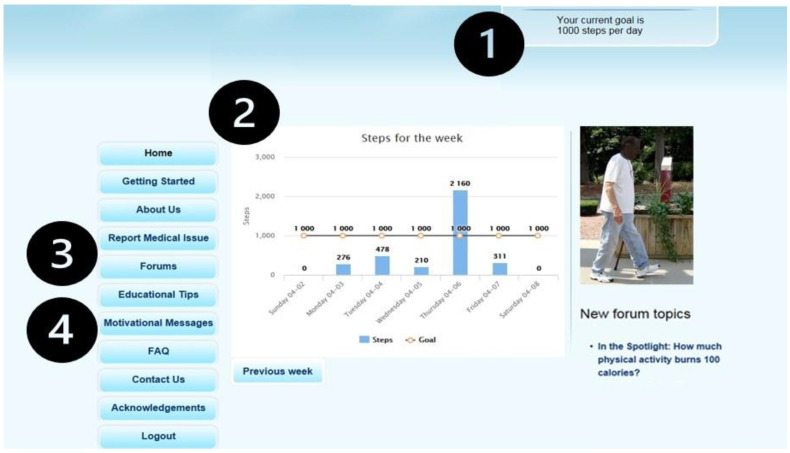
Screenshot of web-based intervention home page. Components include: (1) individualized daily step count goals, (2) iterative feedback on daily step counts objectively measured with a Fitbit, (3) social support via an online forum, and (4) educational tips and motivational messages.

We have developed and rigorously tested a web-based, multicomponent intervention to promote lifestyle physical activity [7,15,16,17,18]. This platform is a dynamic, pedometer-mediated intervention based on the theory of self-regulation, which emphasizes an iterative process to behavior change [19]. The intervention uses four unique components to support individuals with COPD as they learn from their successes and failures to develop effective behavioral strategies to achieve their goals (Figure 1). The intervention components include: (1) individualized step-count goals which are objectively measured with a Fitbit, (2) iterative feedback, (3) an online community for social support, and (4) motivational messages and educational content about managing COPD.

To date, the efficacy of this intervention has been assessed in three randomized controlled trials (RCTs), which compared the impact of the intervention to a control group (pedometer alone or usual care) [15,17,18]. Across all the trials, participants who received the intervention significantly increased their daily step counts compared to the control group [16,17,18]. Those assigned to the intervention have also demonstrated significantly greater improvements in their health-related quality of life (HRQL) [16], and a reduced risk of COPD acute exacerbations [7].

Given the high and growing prevalence of COPD [20], there is an urgent need to leverage implementation science to support the uptake of evidence-based interventions. The practical, robust implementation and sustainability model (PRISM) is a wide-ranging model for translating research into practice. PRISM aids in the assessment of how an intervention is perceived by recipients in order to influence program implementation, reach, and effectiveness [21]. PRISM identifies the factors to consider when translating research into real-world practice, and provides guidance about measuring success and challenges. PRISM can provide a lens through which to evaluate the factors that may influence future implementation efforts, including organizational and patient perspectives, characteristics of the organization and patients, infrastructure of the receiving organization, and the organization’s external environment.

### Current Study

The current study focuses on understanding the patient and clinical stakeholder perspectives regarding the intervention. We aimed to identify the facilitators, barriers, and recommendations that would inform future implementation efforts for a technology-based physical activity intervention for COPD. We leveraged an ongoing RCT of the intervention (COPD Access to PR Intervention (CAPRI); NCT03794921) by using mixed methods to evaluate the patients’ perceptions of the intervention after three months of use (the primary outcome timepoint of the RCT). We also evaluated a diverse sample of clinical providers involved in the care of patients with COPD.

## 2. Materials and Methods

### 2.1. Setting

This convergent, parallel mixed-methods study evaluated patient and provider perceptions of the intervention through semi-structured interviews and surveys, which are detailed below. The data were obtained by a joint research team from two VA Medical Centers within the VA New England Healthcare System (Veterans Integrated Service Network 1). The research activities were approved and monitored by the institutional review board at each participating VA Medical Center. Written informed consent and HIPAA authorization for the use and release of individually identifiable health information were obtained from all the participants prior to enrollment.

### 2.2. Recruitment

#### 2.2.1. Patients

Patients with a diagnosis of COPD who were eligible to participate in the VA Medical Center’s conventional, in-person PR program, but did not, were invited to participate in the RCT. The eligible and interested participants were randomly assigned 1:1 to either usual care or the web-based physical activity intervention. After three months and the completion of the outcomes data collection for the RCT, the participants who were randomly assigned to the intervention were invited to participate in an optional, qualitative feedback interview about their experience using the intervention. All the participants who were invited and agreed to be interviewed provided verbal consent. Recruitment for the interviews continued until thematic saturation was achieved. The sample was recruited and interviewed over a span of 20 months, from January 2020 to August 2021.

#### 2.2.2. Providers

To assess the clinical perspective, we interviewed clinical providers from the organization who interacted with patients with COPD. This included pulmonologists, physician assistants, nurses, sleep technicians, and respiratory therapists. The providers were identified via team discussions with other pulmonologists, the VA Medical Center website, and via snowball sampling. The potential providers were sent an initial recruitment email. Those who did not respond within two weeks were sent a follow-up email. Recruitment continued until we reached thematic saturation in our interviews. A total of 47 providers were sent at least one recruitment email between December 2020 and April 2021. The providers who expressed interest were scheduled to participate in a telephone call or video visit, where their eligibility was confirmed. Providers were considered eligible if they were employed at the VA Medical Center, involved in the direct care of persons with COPD and/or the PR program at the VA Medical Center, had access to the Cisco Webex video platform, and were willing to participate in an audio-recorded interview.

### 2.3. Procedures

#### 2.3.1. Patients

The demographic and clinical characteristics were assessed at a baseline, including the modified Medical Research Council (MMRC) scale, ranging from 0 to 4, where a higher number indicated greater dyspnea [22]. Quantitative surveys that assessed patient perceptions of the usability of the intervention were administered at the 3-month visit. The survey was adapted from the Health Information Technology Usability Evaluation Scale [23] and included four domains: (1) impact, (2) perceived usefulness, (3) perceived ease of use, and (4) user control. Responses could range from 1 (strongly disagree) to 5 (strongly agree). All the items were coded so that a higher number represented greater agreement. We calculated the domain-specific scores (the mean across items within each domain) and an overall score. A higher number represents greater usability.

The first three interviews were conducted in person at the VA Medical Center. In March of 2020, research was paused due to the COVID-19 pandemic. Recruitment resumed in June 2020, and all the remaining interviews were conducted virtually via Cisco Webex. The interviews lasted approximately 30–60 min, were audio recorded, and later transcribed verbatim by a professional transcription service. Two members of the research team trained in qualitative data collection and management (SAR and SKS) conducted all the qualitative interviews. All the participants enrolled in the parent RCT were provided with a $50 payment for each visit, and allowed to keep the Fitbit after their participation was complete. The participants did not receive additional monetary compensation for the optional qualitative interview.

The interviewers used a semi-structured, qualitative interview guide (Supplemental Materials), informed by PRISM, to ask the patient participants about their experiences using the intervention, and to identify factors from the patient perspective that would facilitate or impede the implementation of the intervention. Specifically, we evaluated the patients’ perspectives about the intervention concerning the extent to which the intervention: was patient-centered, provided patients choices, addressed barriers to exercise, seamlessly transitioned between program elements, was accessible, was burdensome to use, supported the development of goals and action plans, and provided patients with feedback.

#### 2.3.2. Providers

All the data collection for the providers was conducted virtually via Cisco Webex. At the start of the video call, the providers were given a survey, which collected information on their demographics and role at VA. They were then given an overview of the intervention, including information on the previous RCTs of the intervention, details on the multiple web-based components (with screenshots), and the evidence to date. After the overview, a semi-structured, qualitative interview guide (Supplemental Materials) was used to ask the providers about their perception of the intervention. PRISM informed the development of the interview guide. Specifically, we evaluated the providers’ perspectives concerning the: strength of the evidence base supporting the intervention, the extent to which the intervention addressed provider-facing barriers, the organization’s readiness to implement such an intervention, the coordination across departments and specialties, the extent to which they would observe the results, and the potential burden and usability of the intervention. After the interview, the interviewer administered a final survey that assessed the providers’ perceptions of the usability of the intervention. The survey was adapted from previous research [24] and was based on the unified theory of acceptance and use of technology (UTAUT) [25,26]. The survey included five domains: (1) behavioral intention to use the intervention, (2) effort expectancy, (3) facilitating conditions, (4) performance expectancy, and (5) social influence. Responses could range from 1 (strongly disagree) to 5 (strongly agree). All the items were coded so that a higher number represented greater agreement. We calculated the domain-specific scores (the mean across items within each domain) and an overall score. A higher number represents greater usability.

### 2.4. Data Analysis

#### 2.4.1. Quantitative Surveys

The survey data were analyzed using descriptive statistics and univariate analysis in SAS 9.4 (Cary, NC, USA).

#### 2.4.2. Qualitative Interviews

The interview transcripts were analyzed utilizing a hybrid approach of deductive and inductive coding and theme development [27,28]. Through intensive discussions, two members of the research team who served as the primary interviewers (SAR and SKS) created two preliminary codebooks, one for the patient interviews and one for the provider interviews. The codebooks contained a priori, deductive codes derived from the PRISM framework and emergent, inductive codes derived from the data. For the patient transcripts, using the preliminary codebook, one transcript was independently coded by both researchers, who then met to compare the coded transcripts, resolve disagreements, and revise the codes and code definitions until a consensus was reached. The two researchers independently coded two additional transcripts with the revised codebook, met to compare the coding and reach a consensus, and further refined the codes and code definitions to create a final codebook. Each researcher then utilized the final codebook to code 6 of the remaining 12 interview transcripts. Upon completion of the coding process, the researchers met to engage in a thematic analysis. The coded data were summarized into an Excel matrix organized using the codes to allow for comparison across the data set, the identification of patterns, the generation of themes, and the examination of the relationships between the themes. This process was repeated for the provider transcripts.

#### 2.4.3. Mixed-Methods Integration

Guided by best practices for mixed-methods research, we integrated the quantitative and qualitative data at the “interpretation and reporting” levels through a narrative and joint display [29]. Integration through a narrative describes the quantitative and qualitative findings in a single report or series of reports [29]. Here, we describe the findings in a single report and use a contiguous approach, in which the quantitative and qualitative findings are reported in different sections within the results [29]. We use a joint display to organize our discussion of the integrated analysis [30].

## 3. Results

### 3.1. Patients

**Figure 2:**
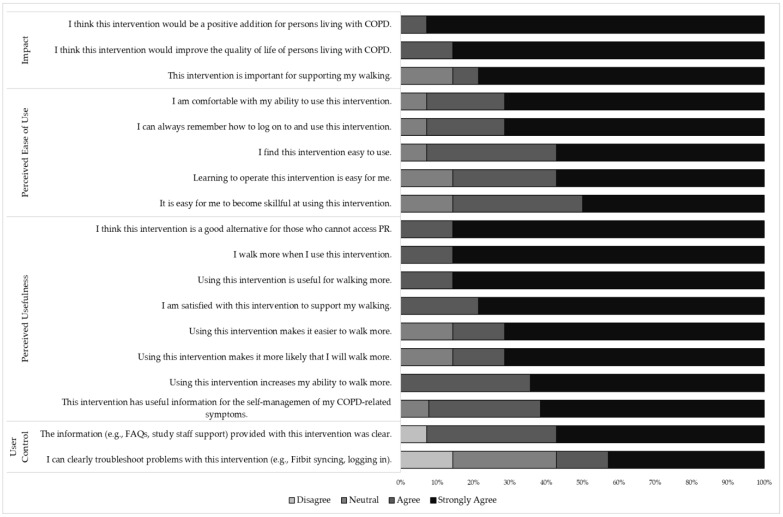
Distribution of patient agreement with intervention usability.

Most of the patients were white (*n* = 13, 87%), all were male (*n* = 15, 100%), had a mean age of 73.5 (SD = 8.0), and mean pack years = 45.4 (SD = 23.48). The patients’ median modified Medical Research Council (MMRC) dyspnea score was 3.00 (IQR = 3). Their detailed sample characteristics are displayed in Table 1. Overall, the patients rated the usability of the intervention as high, with median domain scores ≥4.50 out of 5.00. The overall usability score median was 5.00 (IQR = 1.00). The patients agreed that the intervention was impactful (median = 5.00, IQR = 0.00), useful (median = 5.00, IQR = 0.00), easy to use (median = 5.00, IQR = 1.00), and felt they had a high sense of user control over the intervention (median = 4.50, IQR = 1.25). Figure 2 details the participant responses to the individual survey items.

The key barriers and facilitators identified across the PRISM elements specific to the patient perspective, paired with representative quotes, are summarized in Table 2. Within the interviews, all the patients said that they would recommend the intervention to other veterans with COPD (*N* = 15, 100%). Below, we present the qualitative data from the patient interviews using the PRISM element.

#### 3.1.1. Patient-Centeredness

Patient-centeredness refers to the degree to which the participants felt the intervention was aligned with their preferences and goals for COPD management. The patients detailed various disease management goals, including walking and/or exercising more (*n* = 12, 80%), avoiding shortness of breath (*n* = 6, 40%), and living a longer, high-quality life (*n* = 4, 27%; e.g., being able to spend time with grandchildren). The patients commonly reported that using the intervention supported them in achieving these goals.

Some patients, however, did not find the daily step count goals or website content relevant to their disease management goals. Among the few that reported this, they noted that it limited their engagement with the intervention, as they did not feel it would improve the other outcomes relevant to COPD, such as breathing performance.

#### 3.1.2. Provides Patients Choices

The patients believed the intervention was convenient, as they could access it from home at any time. They agreed that the intervention could provide a good alternative for those who cannot or will not attend in-person programs, noting that the choice to be able to avoid going into the hospital was “very important” because “going to the hospital is a turn off”. However, some noted that this type of remote, self-guided intervention would only be beneficial if the patient has the necessary self-motivation to use it effectively.

All the patients had been eligible to participate in PR but did not. The majority cited the location and time commitment of the program as prominent barriers to attending. It was also noted that the convenience of the intervention would be amenable to busy schedules and competing medical demands. Patients also commented that, now that they had completed this study, they were more interested in participating in a structured PR program. Some were now open to attending conventional, in-person PR programs. Others, who faced major barriers to in-person PR (e.g., distance), expressed interest in interactive, structured virtual PR programs delivered via telehealth.

#### 3.1.3. Addresses Patient Barriers

Patients noted the various intrinsic (e.g., lack of motivation, fear of becoming breathless, and injury/pain) and extrinsic (e.g., bad weather and competing life demands) barriers that impinged upon their daily step count goals. Many felt the intervention supported them in overcoming these barriers. For example, despite difficult weather, many learned from the intervention that they could walk inside large stores to accomplish their daily step count goal. However, some noted that these extrinsic barriers were still difficult to overcome even with the intervention.

#### 3.1.4. Seamlessness of Transition between Program Elements

The seamlessness of the transition between program elements refers to patient perceptions about the various intervention technologies and how they performed together (i.e., Fitbit and website). Most patients reported no trouble using the Fitbit, including using it to track and sync their daily step counts. Some discussed an initial difficulty when learning how to use the Fitbit and sync their data to the website, but were able to overcome any technical issues on their own or by reaching out to the research team. The patients who spent time on the website reported no difficulty navigating to and around the website.

#### 3.1.5. Accessibility

Accessibility denotes patient perceptions of whether they had the appropriate technology and skills to use the intervention. In general, the patients reported that they had the proper technology and experienced little difficulty with this element. However, a couple patients did not have the compatible technology, noting that syncing the Fitbit did not work with their current computer setup.

Additionally, the Fitbit, when worn on the wrist, did not pick up steps when some of the patients were holding on to something for stabilization (e.g., cane, handles on treadmill, or grocery cart), as the wearable captures step counts through arm movement. The participants recommended offering a different device that could track steps for those who cannot move their arms as much when walking, such as a pedometer worn around the waist.

#### 3.1.6. Burden

The burden of the intervention refers to its complexity and patient perceptions regarding the difficulty of using it. Much of the sample reported the intervention was generally easy to use. However, most of the patients seemed to engage primarily with the Fitbit, and approximately half the sample reported not using the intervention website. For those who did access the website, the majority only used it to see their goals and the feedback graph. The patients attributed this lack of engagement with the website to not knowing about the website (*n* = 4), not believing the content was relevant (*n* = 3), or not feeling comfortable using a computer (*n* = 1). The patients believed they would have benefitted from the website more if they were given more hands-on instruction on how to use it or notifications/reminders to access the website.

#### 3.1.7. Developing Goals and Action Plans

Another important element of the PRISM patient perspective is the evaluation of patients’ key barriers and facilitators to developing collaboratively set goals and providing feedback. The majority of patients found the incremental increase in their daily step count goals motivating. However, a few noted that the goals, which were automatically calculated using an algorithm based on patient step data from the prior week, did not meet their expectations. For example, one patient reported being a bit disappointed when there was a smaller change than anticipated. This participant felt that the automated goals were too low, and therefore set his own goals that were higher than those the algorithm set.

#### 3.1.8. Feedback of Results

The patients found great value in the components of the intervention that provided personalized feedback. Regularly receiving results from the feedback graph on the website was motivating. They noted that this type of goal-oriented approach and feedback was reminiscent of their time in service and was motivating. Many also discussed the value of receiving their step-count goal over the phone. While these phone calls were templated calls that only told participants their step-count goal for the next week, the patients reported that these calls provided a sense of accountability and made them feel as though someone cared about their progress and health.

Many thought there would be value to sharing their step data with their clinical team. Additionally, they believed that knowing that their clinical teams were looking at their data to inform their provision of care would be incentivizing to continue walking. The patients did qualify these statements by noting that this would only be motivating if their provider expressed interest in seeing their data and how they were progressing, noting a few past negative experiences when their provider discounted the data they were sharing.

### 3.2. Provider

Fifteen providers participated in this study; their mean age was 49.2 years (SD = 9.8) and 80% (*n* = 120) were female. Most of the sample were pulmonologists (*n* = 6, 40%), 33% (*n* = 5) were nurse practitioners or registered nurses, 13% (*n* = 2) were sleep technicians, and 7% of the sample consisted of a physician assistant (*n* = 1) and a respiratory therapist (*n* = 1). Most reported seeing less than 20 VA patients per week (*n* = 7, 47%) for less than 30 min per visit (*n* = 10, 67%). The majority reported being involved in patient/disease education (*n* = 14, 93%), the nonpharmacological treatment of COPD (*n* = 14, 93%), PR (*n* = 12, 80%), and disease self-management support (*n* = 12, 80%). Regarding the types of technology used in their work, all (*n* = 15, 100%) used electronic medical records, 93% (*n* = 14) used secure messaging, and 73% (*n* = 11) used video calls (VA Video Connect). Detailed provider sample characteristics are displayed in Table 3.

**Figure 3:**
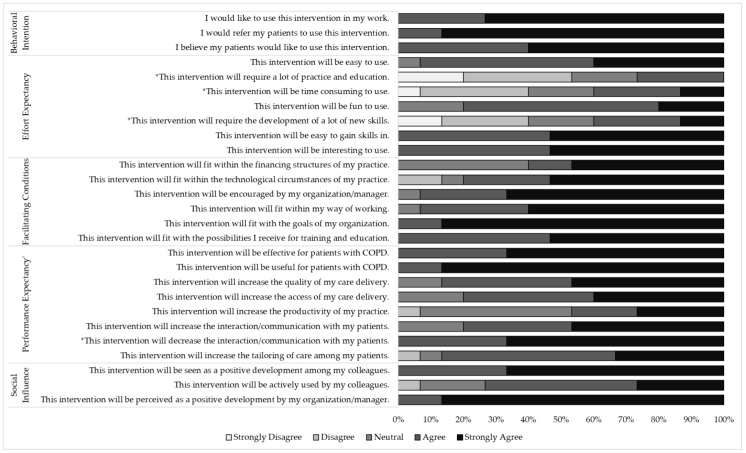
Distribution of provider-participant agreement with intervention usability. * = Raw scores which were reverse coded when calculating summary scores.

Overall, the providers rated the usability of the intervention as moderately high (overall median = 5.00, IQR = 1.00), with all median domain scores ≥ 4.00 out of 5. The providers reported high agreement regarding their intention to use the intervention (median = 5.00, IQR = 1.00), high agreement that their colleagues and organization will have a positive view of the intervention (i.e., social influence: median = 5.00, IQR = 1.00), high agreement that the intervention will fit into their current work environment (i.e., facilitating conditions: median = 5.00, IQR = 1.00), and high agreement that the intervention will be effective in supporting the delivery of care (i.e., performance expectancy: median = 5.00, IQR = 1.00). In comparison to other the domains, agreement was slightly lower for their perception that the intervention would be a low burden to learn and use (i.e., effort expectancy: median = 4.00, IQR = 2.00), though it was still generally positive. Figure 3 displays the mean provider-participant responses to the individual survey items.

Below we present the qualitative data from the provider interviews using the PRISM element. The key barriers and facilitators identified across the PRISM elements specific to the provider perspective are summarized and paired with representative quotes in Table 4. Within the interviews, all the providers (*N* = 15, 100%) said that they would use the intervention in their practice.

#### 3.2.1. Strength of the Evidence Base

Overall, the providers felt confident that the intervention was a “good evidence-based practice” and improved physical activity and other important outcomes relevant to COPD, such as improved HRQL and a decreased risk of acute exacerbations. However, some expressed concern that the participants who enroll in the RCTs may generally be healthier and more motivated than most patients, so there may be some selection bias in the evidence. The providers also noted that there was little evidence of improvements in exercise capacity (i.e., the amount of exercise patients are physically capable of doing), an important target outcome for traditional PR.

#### 3.2.2. Addresses Provider Barriers of Frontline Staff

Providers discussed various patient- and provider-facing barriers to managing COPD. Many discussed the patient-facing access barriers to traditional in-person PR programs. They believed that this intervention could fill an unmet need on the spectrum of the nonpharmacological management of COPD, by providing patients who did not enroll in PR with another alternative. The providers also discussed the barriers to knowing how patients were doing outside of clinical visits. For example, lower daily step counts could reflect a change in medical status.

#### 3.2.3. Readiness and Coordination

All the providers, regardless of their clinical team role, emphasized the importance of integrating the intervention and accompanying data into the electronic health record to avoid having to navigate through other auxiliary platforms to view their patient’s data and progress. Not all provider types agreed that the intervention would fit within their workflow. Non-physician team members (e.g., nurses) more often felt it would complement their existing practice, whereas physicians more frequently felt they would not be able to fit the intervention into their current provision of care. Another barrier identified involved the time it would take to orient the patient to the intervention.

#### 3.2.4. Ability to Observe Results

The providers noted that the intervention’s ability to show changes in daily steps would help inform their care decisions. The providers pointed to other outcomes they would be interested in seeing, like fatigue and dyspnea, and how these fluctuated with the patient’s physical activity. The providers noted that the intervention would offer insight into how much the patient could do, compared to how much the patient is actually doing. Overall, many providers expressed interest in seeing these outcomes aggregated, ranging from weekly progress to a binary snapshot of whether or not they were enrolled. There was not as much interest in seeing the day-to-day fluctuations in data—“I think that we’re all sort of just like tell me what the story is, I don’t need all the other information.” Additionally, some providers noted that some patients may require in-person monitoring when they are exercising, which is not possible with this type of remote, asynchronous intervention.

#### 3.2.5. Burden and Usability

The providers overwhelmingly thought that the intervention seemed user friendly. However, as most of the intended users for this intervention are older adults, it was also noted that age and discomfort with technology may be limiting factors to engaging patients in using this intervention. The providers expressed that comfort with technology was changing, especially as patients become more exposed to technology-based health services. For example, the patients who enroll in virtual PR may feel more comfortable using technology, which might transfer to a greater likelihood of their feeling comfortable using this intervention.

## 4. Discussion

We conducted a convergent, parallel mixed-methods study to evaluate patient and provider perceptions of a web-based, pedometer-mediated physical activity intervention for COPD. The use of quantitative and qualitative components supplemented each other to strengthen the validity of the inferences and to further understand the experiences and perceptions of the intervention. Table 5 is a joint display, which integrates the quantitative and qualitative findings to identify the overall barriers, facilitators, and recommendations for the future implementation of the intervention.

### 4.1. Facilitators

Several facilitators were identified, including: the potential high impact of the intervention on patient health, the usefulness of the intervention to meet an unmet clinical need, and the perceived ease of use of the intervention.

#### 4.1.1. A Potentially High Impact Intervention

Both patients and providers perceived that the intervention had the potential to be highly impactful in supporting COPD management. Notably, all the patients said they would recommend the intervention to another veteran with COPD, and all the providers said they would refer their patients with COPD to the intervention. Through the interviews, we identified that both patients and providers saw value in the intervention for supporting important disease management goals, such as walking more, feeling less short of breath, and having a better quality of life. Physical activity promotion is recommended for all patients with COPD to support these goals [4,31].

#### 4.1.2. Usefulness

The intervention was perceived as useful for fulfilling an unmet clinical need. Specifically, patients and providers alike speculated that the intervention’s flexibility and convenience would made it a suitable better-than-nothing alternative for those who would not access conventional, in-person programs for patients with COPD, like PR. Future work is needed to compare the efficacy of this intervention compared to traditional PR. Patient perceptions regarding the extent to which they feel they are given choices in their care is important to support their sense of autonomy and activation in the management of their health [32]. Therefore, an intervention that provides patients with more choices is likely to support their disease management.

Notably, all the patients interviewed were eligible to participate in a conventional, in-person PR program, but did not. The qualitative interviews with the patients found that, after completing this program, the patients reported being more willing to attend traditional PR or, at least, engage in more activity than before. Furthermore, the providers speculated that the intervention could support the maintenance of benefits acquired during PR. PR is the nonpharmacological standard of care for COPD, but maintenance after the completion of the program is scant. This type of intervention may potentially serve as a gateway either to conventional PR and/or maintenance after PR. Communicating these potential benefits to physicians could ultimately facilitate the adoption of and referral to the intervention.

The providers also speculated that the intervention would be useful for supporting patient–provider communication, and possibly informing clinical care. The ability for patients to collect data outside of a clinic visit and send it to their care team has great potential to provide targeted and efficient care [33]. For example, the ability to see sudden changes in a patient’s activity can alert providers to the worsening of disease status and allow them to intervene before hospitalization is needed [34]. Additionally, tracking daily physical activity can help to support patients’ engagement in their own health (i.e., patient activation), which can support COPD disease management and clinical outcomes [35].

#### 4.1.3. Perceived Ease of Use

Most of the participants believed the intervention was easy to use. The patients who were able to use the intervention for three months reported very few difficulties with the intervention components. Among all the intervention components, the patients commented the most on the automated feedback graphs on the website and the weekly phone call they received from a team member to tell them their new step-count goal. Providing iterative feedback and personalized goals can help to motivate patients and support self-efficacy for engaging in physical activity [36]. While completely automated interventions are the most scalable, interventions that combine and provide both automated and nonautomated aspects (e.g., a phone call from a team member) may be additionally helpful in supporting patient motivation and engagement with the intervention [37].

While the providers had more hesitation about whether this technology-based intervention would be suitable for an older patient population, as is typically seen in COPD, most believed it seemed relatively straightforward. We have also shown, for this particular intervention, scant age-related differences concerning the acceptability of the intervention to patients [38]. There was also strong agreement that their colleagues and organization would see the intervention as a positive addition to their organization.

### 4.2. Barriers

Digital literacy and the fit with current clinical workflows were the main barriers to implementation identified.

#### 4.2.1. Digital Literacy

Despite being introduced to the website after randomization, and the overall perception that the intervention was easy to use, the patients expressed a need for a more deliberate orientation to the website. Some patients may require more detailed instruction and guidance on how to leverage all the components of the website to elicit the most benefits from the intervention. The providers also expressed related concerns about digital literacy in their patient population, which would impede their ability to engage with the intervention. Digital literacy, defined as the interaction of individual and social factors in using digital technologies to “search, acquire, comprehend, appraise, communicate and apply health information” [39] is a common barrier to adopting technology [40,41]. Interventions that embed digital-skills training may help to overcome barriers and facilitate intervention adoption [42,43].

While many patients had a goal to walk more and be more active, some patients had other goals related to their COPD management, which were more focused on their quality of life (e.g., feeling less short of breath and being able to travel more). While physical activity definitively supports HRQL [15,44], some patients did not easily see the connection between physical activity and HRQL. Patients with COPD often report limited understanding and knowledge regarding their self-management [45]. Dedicating time to empower patients with knowledge about the connection between physical activity and other health outcomes may support patient engagement with the intervention [46]. Recent literature suggests that peer-led digital training, where fellow patients train other patients to use the technology, may support digital literacy and increase patient engagement with the intervention by providing technical support and encouraging accountability for continued engagement [42,43].

#### 4.2.2. Fit with Current Clinical Workflow

While the providers acknowledged that spending more time orienting patients to the intervention would be useful, fitting this into their already very demanding workloads was a barrier. The compatibility with workflow was noted as a barrier in two respects: (1) compatibility with current processes and software and (2) compatibility with the scope of their current role. Regarding current processes and software, the providers noted that they would be more willing to engage with the intervention if it was integrated into the electronic health record—a software they already regularly use. Interventions that do not fit into the current clinical workflows present a greater barrier to successful implementation [46]. As a recent and notable example, during the first phase of the COVID-19 pandemic, health care systems faced challenges in adopting technology-based health services, due to an incompatibility between the technology and a clinical workflow that was originally designed for face-to-face care [47,48]. Although technology can be leveraged to lessen a clinical team’s burden, it could have unintended consequences on the working environment if the technology is not properly adapted to the workflow [49].

### 4.3. Recommendations for Future Implementation

Recommendations to inform future implementation efforts include advanced hands-on training and integration of the intervention processes (e.g., referral and monitoring) into the electronic health record. A more thorough orientation to the intervention components would teach patients how to better leverage the multiple components of the website. Additionally, more deliberate training could also be used as an opportunity to help the patient understand the link between physical activity and other important outcomes. If this intervention were to be implemented, PR programs could consider orienting patients to the intervention during one of the PR sessions. Recent studies have found technology-based interventions after the completion of PR to be effective for maintaining activity [47]. For patients who declined to attend PR, they may be more willing to attend one session of PR or virtual PR dedicated to learning about and enrolling in the intervention, as opposed to 8–12 weeks of multiple sessions. To not add to the clinical burden, healthcare systems could also consider leveraging other individuals who are responsible for supporting patient use of technology-based health services, such as connected care coordinators within the Veterans Health Administration. Another promising investment from the VA are virtual health resource centers, which have recently been implemented across several VA Medical Centers [48]. These centers provide a dedicated space and staff with protected time to help orient users to technology-based health services. These types of dedicated resources, either through larger centers or within a specific clinic, would benefit implementation efforts.

Similarly, an effective referral to the intervention, to a coordinator, and/or to a resource center all require a simple process that does not exacerbate the clinical team’s burden. Therefore, the integration of a referral process into the electronic health record will be useful in supporting the implementation of this intervention. Patients strongly value their clinical team’s endorsement of technology-based health services. Recently, a survey of US veterans found that clinical team encouragement was the most important factor to adopting a technology [49]. Enabling a seamless referral process has a higher likelihood of supporting adoption.

### 4.4. Limitations and Future Directions

This study has several limitations. Data were collected from patients who chose to enroll in and completed the RCT. We did not gather data from those who declined to participate or withdrew from the RCT. As such, the patient sample is not necessarily generalizable to all individuals with COPD, particularly as those who declined to participate may perceive technology-based interventions differently than those who participated in this study. Similarly, the responses from the providers represent responses from those who chose to participate in this study. Future work would benefit from evaluating the perceptions of those who declined to enroll, as their perceptions will likely vary from those who felt comfortable and motivated to participate. This study is also limited in its small sample size from one site and may not be representative. A further exploration of patient and provider perceptions across multiple sites in different health care settings could enrich our understanding of the barriers and facilitators, and improve future implementation efforts. The results were based on interviews with and the survey data of patients who recently used the intervention and may be subject to recall bias. Future work might consider leveraging more frequent evaluations of the user experience. Finally, as this study documented a formative evaluation of an intervention not yet implemented in clinical care, the providers were not exposed to using the intervention, nor did they have patients enrolled in the intervention. The providers expressed their perceptions based on what was presented to them. Future work would benefit from larger-scale studies that would gather provider perceptions after implementation at the point of care.

### 4.5. Implications for Practice and Research

The current study provides a meaningful contribution to the literature, in that it uses a theoretically based approach to evaluate the potential barriers and facilitators to a rigorously evaluated and effective technology-based physical activity intervention for COPD [15,17]. This work extends beyond this specific intervention—it also provides insight for future intervention development efforts. There has been an increasing emphasis on the importance of establishing the theoretical bases of interventions and strategies to facilitate implementation [50]. As few technology-based self-management interventions have moved past the pilot phase, it is important to understand the factors that could contribute to the success of the intervention. The field of implementation science is well-positioned to identify these determinants, despite being underutilized within technology-based health research to date [51].

## 5. Conclusions

Patient and provider perspectives are critical when planning to successfully implement a program or intervention. This study used mixed methods to explore patient and clinical team perceptions of a web-based physical activity intervention for COPD. Overall, the intervention was perceived as: having the potential to significantly enhance patient health, being useful in fulfilling an unmet clinical need for many patients with COPD, and easy to use. The barriers included patient digital literacy and the impact on clinical workflow. Future efforts should work to develop strategies that could easily integrate the referral and monitoring process into the clinical workflow, and allocate dedicate time and/or staff to facilitate patients’ understanding of the intervention.

## Figures and Tables

**Table 1:** Patient characteristics (*N* = 15).

Characteristic	*n*	%
Age, Mean (SD)	73.5	8.0
Male	15	100.0
Race		
American Indian or Alaskan Native	1	6.7
Black or African American	1	6.7
White	13	86.7
Ethnicity		
Hispanic or Latino or Spanish Origin	1	6.7
Not Hispanic or Latino or Spanish Origin	14	93.3
Marital Status		
Single, never married	2	13.3
Married or living in a marriage-like relationship	9	60.0
Separated, divorced, or annulled	2	13.3
Widowed	2	13.3
Employment		
Part time	1	6.7
Not working due to disability or illness	4	26.7
Retired	10	66.7
Education		
Did not complete high school	1	6.7
Completed high school	2	13.3
Some college or post high school	8	53.3
Bachelor’s degree or higher	4	26.7
Income		
$15,000–$29,999	5	33.3
$30,000–$49,999	1	6.7
$50,000 or more	9	60.0
Pack Years, Mean (SD)	45.4	23.5
MMRC, Median (IQR)	3.0	3.0

**Table 2:** Patient-facing facilitators and barriers using the PRISM element.

PRISM Domain	Theme	Representative Quotes
Patient-centeredness		
Facilitator	The intervention helped achieve disease management goals to be more active and feel better.	[The step goal] started off low but the more they increased… I took it as a challenge: “can you do it?” Absolutely, I can do it. I got up every day. I challenged myself and I got it done… I feel real good, thank you. And thank you for having the program. Other than that, I’d probably still be sitting home feeling sorry for myself. Now I don’t feel sorry for myself. I get up and I’m doing something positive for myself. And it helps me.—Patient 08, 69 years old
Barrier	Daily step goals were not relevant disease management goals for all the participants.	It would definitely provide an incentive to me if the test said well, you’re breathing at 80% or 60% and at the end of the study, you were breathing 5% higher. Okay, that makes sense to me, let’s continue it.—Patient 07, 72 years old
Provides patients choices to support patient autonomy and activation		
Facilitator	The intervention provided accountability and motivation.	I didn’t have to do it. I’m retired. I’m perfectly willing to sit on the couch and surf the computer and do the, the unphysical activities that I do every day… But having done this program has made me aware that the exercise is something that I want to do as well. So, I don’t have to do it. Now, I want to do it.—Patient 12, 72 years old
Facilitator	The intervention is a suitable alternative for those who cannot or do not want to attend conventional pulmonary rehabilitation.	You’re not a schedule of some kind. You see once in you’re in this age group [laughing] you have a lot of appointments down the road with specialists and so.—Patient 09, 76 years old That’s what [the study] did, it motivated me. And if it wasn’t for that, I would say no, I’m not interested [in pulmonary rehabilitation]… It was the incentive, that was the start of it. Like that was the fuel that you put into the engine.—Patient 14, 81 years old
Barrier	Not all participants will be motivated enough to start the intervention.	It’s a gradual process but it works and it keeps you motivated which I think is the main thing in an exercise program, you need—a lot of times you need that outside motivation to—to get you going.—Patient 06, 75 years old I: Do you think that participating in this program would be helpful for other Veterans with COPD? P: I believe so, yes, if they take it seriously.—Patient 09, 76 years old
Addresses patient barriers		
Facilitator	The intervention overcame intrinsic and extrinsic barriers to physical activity.	You know, like I said, even when it’s too cold, I’ll say, “Come on, let’s go to the grocery store or Walmart or Home Depot”. And we’ll just walk around and look. That way I’m getting my exercise. And it’s a motivating factor from it.—Patient 04, 62 years old
Barrier	Some still struggled to hit step goals given certain extrinsic barriers.	I would prefer to do this in the summertime as opposed to the wintertime. It’s cold. In December, January, February, it gets cold and I didn’t have the incentive to go out and walk as far as I might have in the summer time just because it was cold or snowing.—Patient 07, 72 years old
Seamlessness of the transition between program elements		
Facilitator	The intervention elements synced easily together.	I: Did you have any other issues tracking or viewing your step counts with the Fitbit? P: Not really. I: On [the website]? P: No. —Patient 09, 76 years old
Barrier	Some difficulty syncing and accurately tracking steps.	I found a lot of times difficulty just connecting with that point to where to sync it. It just took me—it would take me quite a while to find the right button to hit to get it to sync. I usually ended up figuring it out on my own.—Patient 06, 75 years old
Accessibility		
Facilitator	Most reported having the proper access to technology.	I: What about specifically, the website or the Fitbit? Did you have any trouble with either of those in terms of kind of tech hiccups? P: No. Except I [laughing] I need my glasses to read it, that’s about all but that’s not a big deal.—Patient 10, 83 years old
Barrier	The intervention did not work with older desktops or phones.	My wife and I finally put in Windows 10 in our computer and our laptop. We knew we had to eventually and we had to jump even faster to get the Fitbit connected.—Patient 03, 74 years old
Barrier	A wrist-worn Fitbit did not accurately capture steps if using an assistive device.	If … you folks had known that I’d fallen and was using a walker then you could have given me a waist pedometer rather than the wrist pedometer. So if some injury to the study person happens to limit use of their arms then they should probably give him something else to measure their steps.—Patient 02, 83 years old
Burden		
Facilitator	The intervention was easy to use.	It worked like clockwork.—Patient 11, 65 years old
Barrier	Participants did not receive enough instruction to take advantage of all of the components of the intervention.	The website, I really haven’t spent much time on it except to find out how many steps I’m supposed to walk.—Patient 09 76 years old I learn much better with hands-on. If I’m sitting next to somebody and they’re showing me what to do as opposed to reading the instructions, you know… I think I would’ve gotten a lot more out of it if I had sat down with somebody and went through the whole process.—Patient 09, 75 years old
Goals and action plans		
Facilitator	The iterative goals were motivating.	I found it helpful that it—it—I’m kind of a goal orientated person. If I set —this is telling me you gotta get 10,000 steps in, well, come hell or high water, I’m gonna get it in… and the best part of it is, is it’s a great reminder because it tells you, you know, either you’re gonna do it or you don’t. I guess that’s the way I am. I just—I like having goals.—Patient 10, 83 years old
Barrier	Sometimes the goals did not match what the participant was anticipating.	I didn’t understand how my weekly count was so low when I did so much. I figured the more I walked the higher my step count would be with you guys… I set my own goals—the first time she called me with my average for the week, it was low so I made myself walk more purposely.—Patient 15, 62 years old
Feedback of results		
Facilitator	The feedback and communication of new daily step goals felt like someone cared about their progress.	It’s showing me that someone is interested in what I’m doing…You know, you have to wonder, you know. Is there anybody else out there that cares about you. And this study has made me feel like there is.—Patient 13, 84 years old I’m probably not the only Veteran that likes getting ‘atta-boys’. And the more you give them—I’m serious because it’s motivating. And being in the service, like you always looked up to your sergeant or your captain and when they gave you an ‘atta-boy’, it really meant a lot. And believe me, that’s how people in the military get motivated and that’s what it’s all about… So when you’re doing a program like this and just getting that phone call once a week just, you know, [research team member] was always kind and always was (saying) “good job, good job, you did a great job” and, you know, it just makes you feel better about doing it.—Patient 15, 62 years old
Facilitator	Believe there is value in sharing step data with their clinical team.	I think if just the provider could see it…they could have a commonality of, “wow [participant name] you did good today” or “how come you were a little off, is there something going on that we should know about.” I feel it’d build a better relationship, more connection.—Patient 03, 74 years old
Barrier	Some did not find the step count to be motivating enough, and wished they were tied to other tests to show proof of improvement.	The last study I went on to, when I had the bone density done, I did get the paper and I shared it with my primary and he just kind of like, didn’t even record it’s like… I said I just thought you might like to see this and he wasn’t really overwhelmed so… I’m not saying all providers are gonna[sic] be like that but it wasn’t his thing that day I guess… It said I had really good show from it, you know, the report was real good, you know. I was kinda[sic] proud of it, maybe, I didn’t understand that either. I don’t know. If I want to share any of this with somebody else I don’t think he’d be the one I was talking to about it.—Patient 11, 65 years old

Note: PRISM = practical, robust implementation and sustainability model.

**Table 3:** Provider characteristics (*N* = 15).

Characteristic	*n*	%
Age, Mean (SD)	49.2	9.8
Gender		
Female	12	80%
Male	3	20%
Role		
Pulmonologist	6	40.0
Nurse Practitioner	5	33.0
Sleep Technician	2	13.0
Physician Assistant	1	7.0
Respiratory Therapist	1	7.0
Typical Number of VA Patients per week		
Less than 20	7	47%
20 to 29	5	33%
30 to 39	1	7%
40 or more	2	13%
Typical Time Spent with Patient during Visit		
Less than 30 min	10	67%
30 min or more, but less than 1 h	3	20%
1 h or more, but less than 2 h	2	13%
Type of Help Offered to Patients with COPD		
Patient/disease education	14	93%
Nonpharmacological treatment	14	93%
Pulmonary rehabilitation	12	80%
Self-management	12	80%
Pharmacological treatment	11	73%
Diagnosis	10	67%
Types of Technology Used in Provision of Care		
Electronic medical record	15	100%
VA Video Connect	11	73%
Secure messaging	14	93%
Clinical Video Telehealth	6	40%
Mobile apps (e.g., MOVE! Coach and Annie)	5	33%
Electronic medical record	15	100%
Awareness of Any Online Self-Management Programs for COPD		
No	10	67%
Yes	5	33%

**Table 4:** Provider-facing facilitators and barriers using the PRISM element.

PRISM Domain	Theme	Representative Quotes
Strength of the Evidence Base		
Facilitator	Felt confident that the intervention improves physical activity and other important outcomes, like a reduced risk of acute exacerbations.	I think it definitely benefits the veteran as far as more steps.—Provider 06, Nurse
Barrier	The sampling bias of healthier, more motivated patients may limit generalizability.	I think that the people who end up participating in a program like this are self-selected, so these are the patients who probably are at baseline, have fewer comorbidities, are more functional, may have…less other psychosocial burdens, you know, the motivational, you know, depression, anxiety, etc. … So I don’t know if you’re sort of selecting out a population of COPD patients that are already primed to do well—Provider 09, Pulmonologist
Barrier	Did not feel confident that the intervention improves exercise capacity.	So, something is better than nothing… Some exercise is better than no exercise, and some goals are better than no goals… I don’t think it would compare to the increase in physical endurance that they get out of [pulmonary rehabilitation] but I do think it’s an alternative.—Provider 06, Nurse
Addresses Provider Barriers		
Facilitator	Fulfills an unmet need in the spectrum of the nonpharmacological management of COPD.	I think it’s able to reach a lot of Veterans who would otherwise not receive any type of intervention… We have the Veterans who are sitting at home… on their couch and they’re not doing anything and they don’t have any direction. So I think this is a great program.—Provider 07, Nurse
Facilitator	Could support patient–provider discussions during visits.	If you had a follow up visit and you could see like what their steps were, their suggested steps were and how many they were doing, it’s a conversation to say okay so they think you could do 5600 steps a day but I noticed you’re only doing 2500…what are the barriers that are keeping you from this? And if they’re saying like oh, I’m getting so short of breath, they think I can do it but I can only do this many, then maybe it’s an opportunity like are you using your… Albuterol because you decide you’re going to exercise, are you waiting, are you taking all your other inhalers appropriately?...Is there some tweaking you might be able to do with either the time of the medication or the actual medication itself.—Provider 02, Nurse
Barrier	Hard to keep patients motivated remotely, and patients need to be motivated to benefit from the intervention.	So I think there’s some advantages but maybe for some people who aren’t quite as motivated, they might fall behind because they don’t have that regular kind of check in other than the weekly call.—Provider 02, Nurse
Readiness and Coordination		
Facilitator	Non-physician clinical team members felt it would fit within their workflows.	I would think probably a respiratory therapist…would be the best [for the referral or enrollment process]—Provider 13, Health Science Specialist
Barrier	Physicians felt it would not fit within their clinical workflow.	As a physician, a clinician, it’s sort of a yes/no for me that they’re doing it. It’s like ones and zeros, that’s all the information I care about. Engagement with it, how they are doing with it that’s the pulmonary rehab sphere. So I don’t necessarily feel like I would want to have onus above it.—Provider 08, Pulmonologist
Barrier	Lack of integration with the electronic health record for referrals and monitoring.	I think if…the provider has to log onto a website that is a potential barrier, that’s like a separate website than the [electronic health record], that could be a potential barrier to kind of see how their patients are doing—Provider 01, Pulmonologist
Barrier	Patients need to be oriented to the intervention.	I am not going to show them how to use it. And so, and I think like literally no one wants to be like showing them how to use an app unless it’s their specific job to do that.—Provider 15, Pulmonologist
Ability to Observe Results		
Facilitator	Being able to see high-level step-goal achievement would help inform care decisions.	But if you knew about what their step goals were and how often they were able to achieve them or surpass them, I think that would be really helpful. Because it also gives us an indication, not only how much are they willing to do but how much can they do. You know, are we being realistic when we’re making medication changes or, you know, deciding all the care. We need to sort of know like what are they—what can they do, what are they willing to do, what are they doing. I think the steps are very important for that.—Provider 02, Pulmonologist
Facilitator	Interested in seeing other outcomes (e.g., quality of life and weight).	I would definitely like to see their steps increasing… Another good thing and this is kind of out there but, you know, it might be good for them to be able to document somewhere … how was their breathing, how were they feeling, how was their fatigue. That’s important.—Provider 06, Nurse
Barrier	Cannot monitor patients in real time.	I think maybe for those people that need the social support or need to be monitored closely when they’re doing, you know, the exercises or whatever, I guess there could be a downside there if they’re not able to be monitored. But I imagine if they’re enrolling in [the intervention] that they probably have been vetted and are deemed safe to do it independently. But I guess that could be one downside.—Provider 02, Nurse
Burden and Usability		
Facilitator	Appears user friendly and very visual.	It seems like it is pretty user friendly which, you know, with the population can be something can be really important.—Provider 09, Nurse
Facilitator	Patients already in virtual programs or using health technology may feel more comfortable using the intervention.	I think maybe the patients that are already in [virtual pulmonary rehabilitation] and are working with the technology, we might have a better chance with those patients rather than someone who hasn’t done this. I think after they’ve done the rehab or once they’re enrolled in rehab and doing a few sessions, we would have a better chance of having people, you know, be interested in [this intervention].—Provider 07, Nurse
Barrier	Hesitations regarding patients’ abilities to use the technology, especially older patients.	The only barrier I think would be… if you have someone that doesn’t know how to use a computer very well and has issues navigating the site. That would be a huge barrier, especially for the older population. We have a lot—a huge older population.—Provider 12, Medical instrument technician

Note: PRISM = practical, robust implementation and sustainability model.

**Table 5:** Joint display of patient- and provider-facing barriers and facilitators to implementing a web-based self-management intervention for COPD.

Participant	Quantitative	Qualitative	Interpretation of Mixed-Methods Findings
Facilitators			
Patient	Impact Median = 5.00 IQR = 0.00 Usefulness Median = 5.00 IQR = 0.00 Easy to Use Median = 5.00 IQR = 1.00	I’m doing something positive for myself. And it helps me.—Patient 08, 69 y.o. If it wasn’t for [the intervention], I would say no, I’m not interested [in pulmonary rehabilitation].—Patient 14, 81 y.o. I did not use the website except for syncing the Fitbit…so that you could see what was going on and just to go back and see that I had…met the goals to see…how many steps I had put in with regard to the goals. So, I did not use the website for anything other than that.—Patient 12, 72 y.o.	All the participants (100%; patients and providers) would recommend and/or refer a patient to the intervention. The patients and providers believed the intervention was relatively straightforward and seemed easy to use. Among those who accessed the website, the patients thought the step-count graphs were most useful. The intervention can improve outcomes, like physical activity and HRQoL, that are both meaningful to the patient and clinically meaningful. The intervention can address many access barriers and was seen as a mechanism to support: activity, motivation, enrollment in pulmonary rehabilitation, and patient–provider communication.
Provider	Intention to Use Median = 5.00 IQR = 1.00 Social Influence Median = 5.00 IQR = 1.00	I think it’s great—Provider 04, Physician Assistant I think it definitely benefits the Veteran as far as more steps—Provider 06, Nurse This is something that they could continue that it could be ongoing where they can log on. I think it would be really beneficial.—Provider 07, Nurse I think it could be motivational for some patients that are willing to make the change, who are willing to put in the effort.—Provider 13, Health Science Specialist	
Barriers			
Patient	User Control Median = 4.50 IQR = 1.25	I just remember when I first started just trying to get the Fitbit set up was a little difficult, but it worked out.—Patient 08, 69 y.o. I think I would’ve gotten a lot more out of it if I had sat down with somebody and went through the whole process.—Patient 06, 75 y.o. Some… concrete measure is important to me. Without really knowing what the end result is, I’m not sure of the motivation.—Patient 07, 72 y.o.	The patients noted some initial difficulties getting the technology set up, and did not take full advantage of the multiple intervention components on the website. Some of the patients and providers wished there were improvements to more standard outcomes, like exercise capacity. The providers were more hesitant about the burden it would place on their already very heavy clinical load. There remain some barriers to support that the intervention could not address, such as prioritizing other health issues, monitoring patients in real time, and weather.
Provider	Performance Expectancy Median = 5.00 IQR = 1.00 Effort Expectancy Median = 4.00 IQR = 2.00 Facilitating Conditions Median = 5.00 IQR = 1.00	You don’t have a provider there helping and watching and monitoring for any symptoms that they may be having.—Provider 11, Nurse Whoever runs the PR program (respiratory therapist, licensed therapist, kinesiotherapist) would be better suited for monitoring this information, not necessarily the referring provider.—Provider 01, Pulmonologist If there is[sic] multiple steps and things to fill out, I probably would be less inclined [to refer someone to the intervention] especially because I imagine that some of this role will fall on me as the NP just because that’s kind of the way things happen sometimes.—Provider 02, Nurse	
Recommendations			
Provide a more thorough orientation to the website and technology to support patients’ use of the multiple intervention components. Integrate the referral and display of patient outcomes into the electronic health record. Identify specific team members, or external resources, with the bandwidth to support referrals and the monitoring of patients while enrolled.			

## Data Availability

The data are not publicly available due to the Department of Veterans Affairs data safety and privacy regulations.

## Supplementary Materials

The following supporting information can be downloaded at: https://www.mdpi.com/article/10.3390/jcm12196296/s1, Table S1: Patient Interview Guide; Table S2: Provider Interview Guide.
