# Supplementary material for: Stakeholder Perceptions of a Web-Based Physical Activity Intervention for COPD: A Mixed-Methods Study
Source: J Clin Med. 2023 Sep 29;12(19):6296. doi: 10.3390/jcm12196296 (PMC10574016; doi:10.3390/jcm12196296)
Supplement: Supplementary file 1 [file jcm-12-06296-s001.zip › jcm-2590405-supplementary.pdf]

Supplement

Patient Semi-structured Interview Guide

Patient-centeredness

- What are some goals that you have for managing your COPD? (e.g., walk more, feel less short of breath, do more activities that I used to do, less hospital visits, etc.)
    - What are some things that you do to help you reach those goals?
    - What are some things that make it difficult for you to achieve those goals?
    - Do you feel like the website and Fitbit helped you achieve those goals? How so?
- Tell me about your experience using the website and Fitbit. Was using the website and Fitbit helpful?
    - If so: There were some different parts of the website.
        - motivational messages
        - educational tips
        - online forum
        - the ability to see your steps and get feedback about how you are doing and receive concrete step-count goals.
    - Which parts did you find the most helpful? Why?
    - If no: Why not? What would make it more helpful?
- Now, you mentioned that you found ________helpful (refer to parts of website/Fitbit patient found helpful from beginning). Were there any aspects of the website and Fitbit that you didn't find helpful? How come?
    - Probe (Fitbit, Tips, Motivational Messages, Personalized Goals, Feedback, Forum)
- You mentioned _______ as a goal for managing your COPD (refer to beginning). Can you think of any changes you would make to the website and Fitbit so that they would do a better job to support your goals for your walking and management of COPD?
- Did the website and Fitbit motivate you to walk more?
    - If so: Which aspects motivated you the most? (probe: tips, motivational messages, personalized goals, feedback)
    - If no: Why not? What would make it more motivating?

- Were the tips and messages easy to understand? Did they relate to your experience with COPD?

- You received feedback about your daily activity levels in a couple of different ways. One was directly on the website, and the other was when you received a call from Paola or Lizzy about your weekly step-count goals. Did you find these helpful? Was one more helpful than the other? **(addresses feedback of results)**

Service and Access/Seamlessness between Program Elements

- Did you have any issues tracking or viewing your step counts and activity with your Fitbit and the website?

- You used the website and Fitbit as part of a research study. If in the future, your healthcare provider could use this in the clinic, is there any information from the website and Fitbit that you think would be helpful for your COPD provider or healthcare team to see?

Address Patient Barriers

- Have you ever been referred to, or has your doctor ever discussed pulmonary rehabilitation with you?? It takes place in Jamaica Plain, typically involves coming to the hospital 2-3 times a week for about 8 – 12 weeks.

    - If yes, did you attend? Why/Why not?

        - Each person has his own reasons that make it difficult to attend. Can we talk about what some of those reasons were for you?

        - Probe: lack of motivation, transportation problems, COPD exacerbation, work-related reasons, caretaker responsibilities, fear of getting sicker, think too frail to exercise etc.

- Now that you've participated in the study, if you were referred to a hospital-based pulmonary rehabilitation program by your healthcare provider, would you go?

    - Why/Why not?

    - Has your participation in the study affected whether or not you think you'd attend?

- You've put in all of this hard work to try and increase your walking and moderate intensity exercise. Now that your participation in the study is over, do you have any plans to continue walking and being active?

    - If so, what are some strategies you'll use to help you stay active? (Probe: Fitbit, other exercise apps, etc.

- Comparing the website and Fitbit to what you know of pulmonary rehabilitation, what do you think of having the choice to use the website and Fitbit as an alternative if you can't come to the hospital?

  - Would you recommend the website and Fitbit to other Veterans with COPD?

Provider Semi-structured Interview Guide

Impression of Intervention and Comparison with Conventional Pulmonary Rehabilitation

First, I would like to discuss your overall impression of the intervention. I would also like to discuss your views of what works well, and what doesn't work so well, in the context of care for patients with COPD, including conventional pulmonary rehabilitation and how you feel the intervention might compare.

- What is your overall impression of the intervention?
  - What do you think of the evidence base presented in the slideshow for using this intervention to enhance physical activity in patients with COPD?
- Can you tell me about your experience with conventional, hospital-based pulmonary rehabilitation?
  - How often would you say your efforts to use conventional pulmonary rehabilitation for patients with COPD been effective? (e.g., if you refer patients to PR, do they attend/adhere? If you conduct PR, do patients attend/adhere? Do they improve clinically?)

  If no direct experience:

  - Can you tell me about your thoughts on conventional, hospital-based pulmonary rehabilitation based on what you may have heard about it through your job?
- What do you feel are the important elements of conventional pulmonary rehabilitation that can make it beneficial for patients with COPD?
  - You identified […….…] as important elements of conventional pulmonary rehabilitation. Do you think the intervention could also address […….…]?
  - Are there any elements of conventional pulmonary rehabilitation that you think the intervention fails to address?

  [If participant identifies a new aspect of conventional pulmonary rehabilitation here, interviewer should clarify that the participant feels it is an important element of conventional pulmonary rehabilitation and add it to the notes]

We've talked about some of the things that work well with conventional, hospital-based pulmonary rehabilitation and how the intervention compares on those points. Now I'd like to talk about some of the things conventional pulmonary rehabilitation may not do so well.

- What do you feel are some of the shortcomings of conventional pulmonary rehabilitation? *[Note: If participant has already mentioned some shortcomings, interviewer should acknowledge these.]*

- You identified [………] as shortcomings of conventional pulmonary rehabilitation. Do you think the intervention could address any of these shortcomings? If so, which?

- What do you think would be the shortcomings of using the intervention?

- What barriers do you think impede patients from accessing traditional pulmonary rehabilitation?

  - You identified [………] as barriers to conventional pulmonary rehabilitation. Do you think the intervention could address any of these barriers? If so, which?

  - What do you think would be the barriers to using the intervention?

Thank you for sharing with me a little about what you perceive to be the barriers to both conventional pulmonary rehabilitation and the intervention. Now I would like to ask you about facilitators for using these interventions.

- What aspects of conventional pulmonary rehabilitation do you think help encourage patients to enroll and attend?

- Are there any aspects of the intervention that you think might be helpful to encourage patients to enroll and use it?

COVID-19

- Obviously, COVID-19 has had significant consequences on conventional pulmonary rehabilitation. Can you tell me about your experience with conventional pulmonary rehabilitation during the COVID-19 pandemic?

- Are you aware of any efforts to adapt conventional pulmonary rehabilitation to meet COVID-19 restrictions?

[If participant is aware of adaptations, probe further]:

- How do you think these adaptations may affect the important aspects you detailed earlier? ***[remind participant of what important elements they brought up]***

- Do you think these adaptations help to overcome some of the barriers of conventional pulmonary rehabilitation?

- Do you think these adaptations add any new barriers to patients' access to pulmonary rehabilitation services?

- I'm interested in hearing how you think these three potential services compare: conventional pulmonary rehabilitation, COVID-19 adapted pulmonary rehabilitation **[if they were aware of any]**, and the intervention?

[If participant is not aware of any changes, proceed to next section]

Future Implementation of the Intervention at VA Facility

Thank you for talking with us about the intervention and how it compares to conventional pulmonary rehabilitation. Now we want to talk with you about the potential future implementation of the intervention here. We want to begin by talking about how the intervention could assist you with the management of your patients, how it could fit into your current workflow, and how the referral process might look for you.

- What kind of information would you like to see to assist with the management of your patients? (e.g., steps, logons to the website, what they've viewed on the site)
- When and how often would you want to see it? (e.g., summary, raw data, continuously, between visits, post-exacerbation and/or hospitalization?)
- What would be the best method to provide this information to you? (e.g., dashboard, clinical note)
- Would you be willing to monitor this? If not, who would you want to monitor this?
- Based on what we've talked about today, would you refer a patient to use the intervention? Why/Why not?
- Is there any other information that you would like to see before referring your patients to the intervention?
- Do you think your patients would feel comfortable using the intervention?
- Are there certain patient characteristics that you think would make some patients feel more comfortable using this than others?
- What potential barriers do you foresee when referring patients to use the intervention?
- Can you think of some facilitators, or things that would enable you as the provider to refer patients to this intervention?

Wrap-up

- Thank you for answering these interview questions. Before we transition to final part of the study visit, the last quick questionnaire, is there anything else you would like to tell me about the intervention that I have not asked?
- Can you think of any other providers or employees with whom we should speak?
